# Red Light Enhances the Antioxidant Properties and Growth of *Rubus hongnoensis*

**DOI:** 10.3390/plants10122589

**Published:** 2021-11-26

**Authors:** Hyeon Eui Oh, Ara Yoon, Yoo Gyeong Park

**Affiliations:** National Institute of Biological Resources (NIBR), 1008-11, Sangnam-ro, Sangnam-myeon, Miryang 50452, Korea; hy1906@korea.kr (H.E.O.); yoonara@korea.kr (A.Y.)

**Keywords:** antioxidant activity, blue LED, photomorphogenesis, photosynthesis, red LED

## Abstract

The purpose of this study was to determine the effect of light quality on *R.* *hongnoensis* growth, physiology, and antioxidant properties. Five light conditions were employed, including white (control), red (R), blue (B), combined LED of R, green (G), and B at 7:1:2 (RGB), as well as combined LED of R, G, B, and far-red (Fr) at 7:1:2:1 (RGBFr). R light had the greatest growth-promoting effect based on plant height, leaf length, leaf width, stem diameter, and leaf area. However, leaf width and root length exhibited the greatest growth under RGB. The fresh and dry weight of shoots and roots were highest under R and RGB light. Photosynthesis was highest under RGB and lowest under B. Transpiration was highest in RGBFr. Stomatal conductance and photosynthetic water use efficiency were greatest under RGBFr. Total phenol content and radical scavenging activity were highest under R, while total flavonoid content was highest under RGB. Superoxide dismutase (SOD), catalase (CAT), and ascorbate peroxidase (APX) activities were upregulated under W, whereas guaiacol peroxidase (GPX) activity was highest under RGB. The present results suggest that, among the tested light treatments, R light was most conductive for vegetative growth and antioxidant capacity in *R. hongnoensis*.

## 1. Introduction

Among Rubus species, the thornberry (*Rubus hongnoensis* Nakai) is an endemic plant belonging to the subgenus Idaeobatus and sect Rosaefolii [1]. It was first collected and identified as a new species by Nakai from a region called Hongno (near Cheonjiyeon Falls, Seogwipo), which is located on Jeju Island [2]. Rubus species have been used in traditional medicine for their many medicinal properties [3]. Components isolated from representatives of the genus have been reported to exhibit various biological activities, including antioxidant, anti-inflammatory, antibacterial, and anticancer activity [4]. As a result of comparing the physiological properties of 26 wild Rubus plants (leaf), some species showed high phenolic compound content and antioxidant activity, suggesting potential use as medicine or herbal tea [5]. Wild Rubus plants (fruits) can be used as an energy source because of their high content of essential minerals and carbohydrates [6].

Light quality can indirectly affect biomass production by influencing plant morphology, architecture, and photosynthesis [7]. Red (R) light plays an important role in photosynthesis as well as the control of shoot weight, stem diameter, and leaf area [8]. Blue (B) light not only affects plant growth, leaf expansion, and stomatal opening, but also enhances chlorophyll, flavonoid, and total phenolic content as well as antioxidant capacity [9]. The combination of R and B light is optimal for the growth and development of cucumbers [10]. Although the underlying mechanism of plant growth promotion via green (G) light is unclear [11], it has been reported that the addition of G to R and B light promotes the growth of lettuce [12] and induces an effect similar to that of shade avoidance [13]. Far-red (Fr) light has a higher leaf transmittance than R light. Therefore, it is possible to produce uniform seedlings under Fr light, as it contributes to a lower variation of seedling size [14]. However, despite the many studies above, there are no studies on the effects of various light-emitting diode (LED) light sources on the growth and antioxidant activity of *R. hongnoensis*.

In this study, we hypothesized that applying R, B, G, and Fr as monochromatic light or in combination would influence gas exchange, antioxidant activity, and the growth of *R. hongnoensis*. Therefore, the effects of four different LED spectra on growth, photosynthesis, and antioxidant activity of *R. hongnoensis* were investigated. R light considerably promoted plant growth as well as antioxidant capacity.

## 2. Results

**Figure 1:**
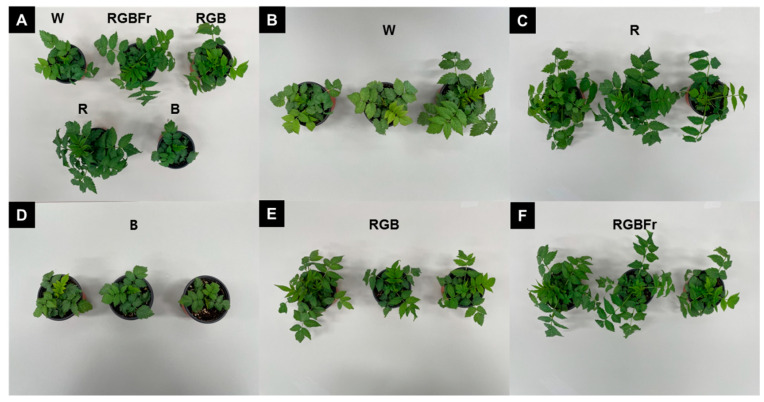
Comparison of shoots and roots of *Rubus hongnoensis* grown under different light-emitting diode (LED) treatment over 8 weeks: Overall view (**A**), white (**B**), red (**C**), blue (**D**), combined LEDs of R, G, and B at 7:1:2 (**E**), and combined LEDs of R, G, B, and Fr at 7:1:2:1 (**F**).

Plant height was greatest under R light (11.2 cm), the leaf length was 16.7 cm, leaf width was 10.4 cm, and the leaf area was 66.2 cm^2^, which altogether indicated a great improvement in leaf growth when compared to parameters under other light treatment conditions (Table 1, Figure 1). Leaf length, leaf width, and leaf area were the lowest under B light (Table 1). The number of five-leaflets was highest under RGBFr light (Table 1). Chlorophyll content was greatest under R (40.7), but no significant difference was determined between treatment groups (Table 1). Stem diameter was greatest under R and lowest under B light (Table 1).

Root length (38.4 cm) was significantly higher under RGB than under the other treatment conditions (Table 2). The fresh weight (8.59 g) and dry weight (0.66 g) of roots were highest under R and RGB light, but there were no significant differences between groups (Table 2). The fresh weight and dry weight of shoots were similar between R (fresh weight-11.65 g; dry weight-2.05 g) and RGB light (fresh weight-11.9 g; dry weight-1.94 g) (Table 2).

**Figure 2:**
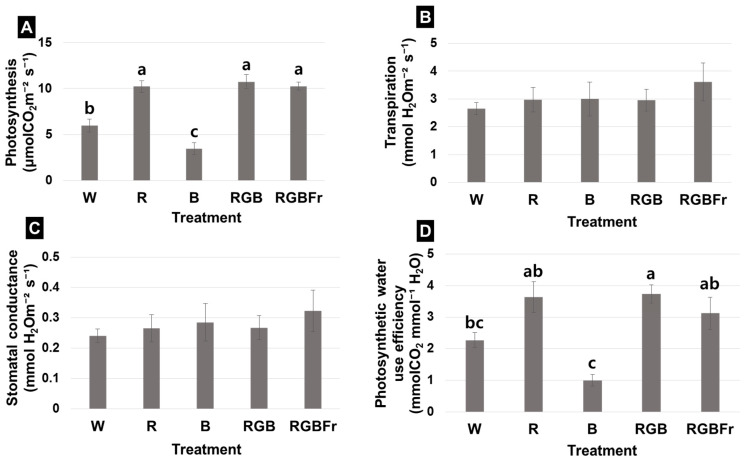
Effect of different light-emitting diode (LED) quality on photosynthesis (**A**), transpiration (**B**), stomatal conductance (**C**), and photosynthetic water use efficiency (**D**) in the leaves of *Rubus hongnoensis*. Light quality applied included W, white (as the control) light-emitting diodes (LEDs); R, red LEDs; B, blue LEDs; RGB, combined LEDs of R, green (G), and B at 7:1:2; and RGBFr, combined LEDs of R, G, B, and far-red (Fr) at 7:1:2:1. Data are the mean ± S.E of the 5 biological replicates. Means accompanied by different letters are significantly different (*p* < 0.05) according to the Duncan’s multiple range test at 5% significance level.

Photosynthesis was highest under RGB (10.74 μmol CO_2_ m^−2^ s^−1^), followed by RGBFr (10.26 μmol CO_2_ m^−2^ s^−1^) and R light (10.22 μmol CO_2_ m^−2^ s^−1^), while being lowest under B light (3.44 μmol CO_2_·m^−2^ s^−1^) (Figure 2A). Interestingly, photosynthesis was enhanced under exposure to R light, as observed for R, RGB, and RGBFr treatment (Figure 2A). Transpiration and stomatal conductance were highest under RGBFr and lowest under W light (Figure 2B,C). Photosynthetic water use efficiency was highest under RGB, followed by R and RGBFr (Figure 2D). Photosynthetic water use efficiency was lowest in B (Figure 2D).

**Figure 3:**
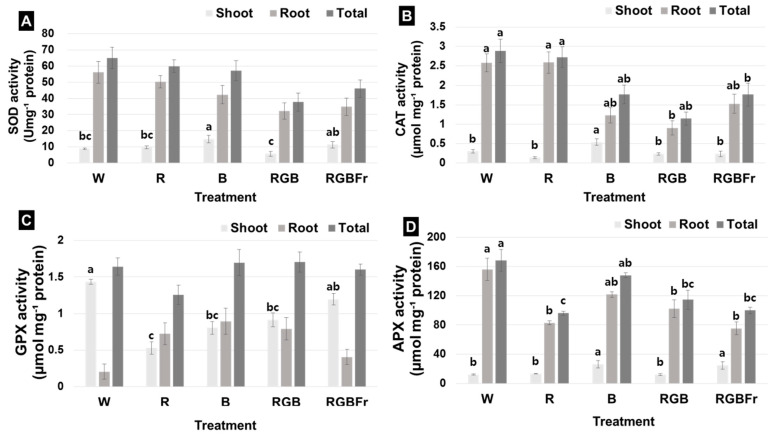
Superoxide dismutase (SOD) activity (**A**), catalase (CAT) activity (**B**), guaiacol peroxidase (GPX) activity (**C**), and ascorbate peroxidase (APX) activity (**D**) of *Rubus hongnoensis* as affected by light quality. Light quality applied included W, white (as the control) light-emitting diodes (LEDs); R, red LEDs; B, blue LEDs; RGB, combined LEDs of R, green (G), and B at 7:1:2; and RGBFr, combined LEDs of R, G, B, and far-red (Fr) at 7:1:2:1. Data are the mean ± S.E of the 5 biological replicates. Means accompanied by different letters are significantly different (*p* < 0.05) according to the Duncan’s multiple range test at 5% significance level.

Superoxide dismutase (SOD) activity of shoots was highest under B (14.9 Umg^−¹^ protein), followed by RGBFr (11.3 Umg^−1^ protein) and R light (9.7 Umg^−1^ protein) (Figure 3A). SOD activity of the root was highest under W (56.2 Umg^−1^ protein) and lowest under RGB (32.1 Umg^−1^ protein) (Figure 3A). Total SOD activity was the highest under W (65.1 Umg^−1^ protein) (Figure 3A).

Catalase (CAT) activity in shoots was the highest under B (0.538 μmol mg^−1^ protein), while that in roots was highest under W light (2.584 μmol mg^−1^ protein) (Figure 3B). Total CAT activity was highest and similar between W (2.888 μmol mg^−1^ protein) and R (2.728 µmol mg^−1^ protein), followed by B light (1.766 µmol mg^−1^ protein) (Figure 3B).

Guaiacol peroxidase (GPX) activity of shoots was highest under W (1.435 μmol mg^−1^ protein), followed by RGBFr (1.195 µmol mg^−1^ protein), RGB (0.914 µmol mg^−1^ protein), B (0.804 µmol mg^−1^ protein), and R light (0.530 µmol mg^−1^ protein) (Figure 3C). GPX activity in the root was lowest under W light (0.207 µmol mg^−1^ protein) (Figure 3C). The total GPX activity was highest under RGB light (1.705 μmol mg^−¹^ protein) (Figure 3C).

Ascorbate peroxidase (APX) activity in the shoot was similarly high under B (26.4 μmol mg^−1^ protein) and RGBFr (24.5 µmol mg^−1^ protein), followed by R (13.3 µmol mg^−1^ protein), RGB (12.2 µmol mg^−1^ protein), and W treatment (12.1 µmol mg^−1^ protein) (Figure 3D). APX activity of the root was highest under W (156.1 μmol mg^−1^ protein) as was APX activity (227.7 µmol mg^−1^ protein) (Figure 3D).

**Figure 4:**
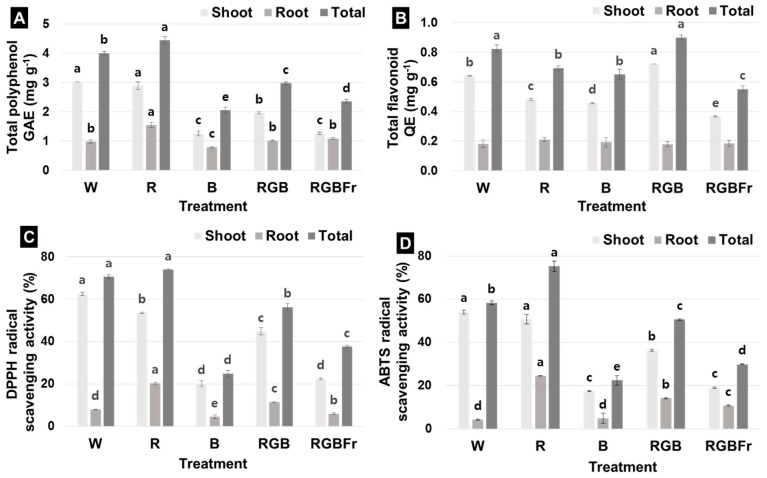
Total phenol content (**A**), total flavonoid content (**B**), 2,2-Diphenyl-1-picrylhydrazyl (DPPH) radical scavenging **(C)**, and 2,2’-azinobis-(3-ethyl-benzothiazoline)-sulfonic acid (ABTS) radical scavenging activity (**D**) in *R. hongnoensis* was affected by light quality. Light quality applied included W, white (as the control) light-emitting diodes (LEDs); R, red LEDs; B, blue LEDs; RGB, combined LEDs of R, green (G), and B at 7:1:2; and RGBFr, combined LEDs of R, G, B, and far-red (Fr) at 7:1:2:1. Data are the mean ± S.E of the 5 biological replicates. Means accompanied by different letters are significantly different (*p* < 0.05) according to the Duncan’s multiple range test at 5% significance level.

Total phenol content of the shoot was the highest under W (3.03 mg g^−1^), followed by R, RGB, RGBFr, and lowest under B light (1.26 mg g^−1^) (Figure 4A). In the root, phenol content was highest under R (1.547 mg g^−1^) and lowest under B light (0.75 mg g^−1^) (Figure 4A). Total phenol content was highest under R (4.44 mg g^−1^), followed by W (4.01 mg g^−1^), RGB (2.98 mg g^−1^), and RGBFr (2.36 mg g^−1^), again being lowest under B light (2.05 mg g^−1^) (Figure 4A).

Total flavonoid content of the shoot was the highest under RGB (0.72 mg g^−1^), followed by W (0.64 mg g^−1^), R (0.48 mg g^−1^), B (0.46 mg g^−1^), and RGBFr light (0.37 mg·g^−1^) (Figure 4B). Root flavonoid content was highest under R (0.21 mg g^−1^), but there was no significant difference between treatment groups (Figure 4B). Total flavonoid content was higher under RGB (0.9 mg g^−1^) than W (0.82 mg g^−1^) and lowest under RGBFr light (0.55 mg g^−1^) (Figure 4B).

The 2,2-Diphenyl-1-picrylhydrazyl (DPPH) radical scavenging activity of shoots was highest under W (62.6%), followed by R (53.6%), RGB (44.8%), RGBFr (22.5%), and lowest under B light (20.2%) (Figure 4C). In contrast, DPPH activity in roots was highest under R (20.3%) and lowest under B (4.6%) (Figure 4C). Total DPPH activity was highest under R (73.9%), followed by W (70.6%), RGB (56.3%), RGBFr (37.6%), and lowest under B light (24.8%) (Figure 4C).

The 2,2′-azinobis-(3-ethyl-benzothiazoline)-sulfonic acid (ABTS) radical scavenging activity of shoots was highest under W (54.2%), followed by R (50.7%), RGB (36.4%), RGBFr (19.2%), and lowest under B light (17.5%) (Figure 4D). Root ABTS activity was highest under R (24.6%) and lowest under W (4.2%) (Figure 4D). Total ABTS activity was highest under R (75.3%), followed by W (58.4%), RGB (50.6%), RGBFr (29.9%), and lowest under B light (22.5%) (Figure 4D).

## 3. Discussion

### 3.1. Morphogenesis

In the present study, leaf growth parameters, such as length, width, and leaf area, were highest under R as compared to the control treatment. Ohashi-Kaneko et al. [15] found that *Brassica campestris* leaf growth was also greatest under R light. According to Wu and Lin [16], *Protea cynaroides* plantlets grown under R LEDs produced a significantly higher number of new leaves compared to those grown under other LED treatments. The fresh and dry weights of *R. hongnoensis* shoots were similar under R and RGB light conditions in the present study. Heo et al. [17] reported that the fresh and dry weights of grape increased under R light. Lee et al. [18] demonstrated the shoot and root growth-promoting effect under different light conditions, including R light, which was also observed in the present experiments.

Pecháčková [19] noted that root growth and development can be altered by light quality. Root growth was enhanced under R, RGB, and RGBFr treatment in the current study, indicating that light sources containing R had a favorable effect on this parameter. Similar observations were reported in *Gossypium* *hirutum* [20], and *Chrysanthemum morifolium* [21], where R LEDs were found to stimulate root formation. According to Wu and Lin [16], *Protea cynaroides* root growth was highest under R light. Simlat et al. [21] also reported that R light had a positive effect on root growth.

In the present study, all evaluated growth parameters of *R. hongnoensis* were lowest under B, suggesting that B light did not promote growth. A similar result was reported by Heo et al. [17] who demonstrated the growth inhibitory effect of B light in the sprouts of some greenhouse crops. In addition, Wu et al. [22] reported that the elongation of *Solanum lycopersicum* was inhibited by B light.

### 3.2. Photosynthesis

Light provides energy for photosynthesis, and thus light quality has major influence on the process [9]. In the present study, photosynthesis was greatest under RGB compared the control W light condition, followed by RGBFr and R, while B light treatment resulted in the lowest photosynthetic activity. Similar results were reported by Kim et al. [12], who demonstrated that RGB treatment enhanced lettuce growth. Although the combination of R and B LEDs has great potential use as a light source for enhancing photosynthesis, plants have adapted to utilize a wider spectrum of light to control photomorphogenesis [23]. Emerson and Rabinowitch [24] reported that photosynthesis was enhanced under the concurrent application of two light beams of different quality.

The transpiration rate and stomatal conductance increased under RGBFr, RGB, and B light in the present study. A similar result was reported by Yorio et al. [25], who reported that stomatal opening was stimulated in the leaves of lettuce grown under R LED light supplemented with B light. B light strongly affects plant growth and development, including leaf size, stomatal opening, and photosynthesis [26]. In the present study, the photosynthetic efficiency of *R. hongnoensis* leaves was improved under all light sources containing R light, while monochromatic B light had a negative effect on photosynthesis. Similar results were reported that R light was important for photosynthetic apparatus development as it enhanced starch accumulation in various plant species by inhibiting the translocation of photosynthates out of leaves [27,28].

### 3.3. Antioxidant

Light is known to affect not only plant growth and development but also the biosynthesis of primary and secondary metabolites [29]. Various LED radiation treatments have been reported to promote antioxidant enzyme activity [21]. In the present study, we also observed that different LED light treatment affected the activity of reactive oxygen species-scavenging enzymes in *R. hongnoensis*. SOD, commonly called metalloenzyme, decomposes two highly reactive O_2_^−^ to produce H_2_O_2_ and O_2_ [30]. CAT, APX, and GPX reduce H_2_O_2_ to water and molecular oxygen [30]. In *R. hongnoensis* shoots, the production of H_2_O_2_ and O_2_ increased as the SOD activity increased under B light, and the CAT and APX activities were also increased to degrade the increased H_2_O_2_. B LED treatment was promoted on ROS-scavenging enzyme activity (SOD, CAT, and APX), opposite to that of R treatment [31]. It is interesting that the GPX activity of root under W and RGBFr is lower than shoot. Although the lower dry weight of the roots under the W treatment might suggest that the root growth in the container was less stressful, it is not clear why the GPX activity was lower in the RGBFr treatment.

In addition to antioxidant enzymes, plants produce various antioxidant compounds, such as phenols, in an attempt to respond and adapt to various biotic as well as abiotic stressors that would otherwise damage the photosynthetic apparatus [32]. Phenols are abundant secondary metabolites in plants and act as natural antioxidants with a wide range of biological activities, including antioxidant, anticancer, antibacterial, and anti-inflammatory activities [33]. In the present study, B light clearly suppressed the accumulation of phenol contents in *R. hongnoensis*. Zheng and Van Labeke [34] reported that total phenol content in the leaves of *Dendranthema morifolium* was suppressed under B light, although this effect depended on the cultivar. B, R, and FR wavelengths also regulate the biosynthesis of phenol contents in a direct or indirect manner through signaling, which leads to the expression of key enzymes, or through upregulating the production of shikimic acid, which is a precursor of phenol contents [35]. Moreover, the activity of phenylalanine ammonia lyase (PAL), a major enzyme involved in phenolic biosynthesis, is known to be regulated by light quality [36]. The total phenol content of shoots and roots in the present study was highest under R, being higher in shoots. R LEDs have been widely employed as an alternative source of illumination for in vitro survival as well as for enhancing metabolite production in medicinally important plants [37]. Shohael et al. [38] demonstrated that R light induced the synthesis of secondary metabolites in *Eleutherococcus senticosus*, resulting in the highest total phenol content, as opposed to B light under which phenol content was lowest.

Plant growth and flavonoid biosynthesis are stimulated by multiple factors, including the specific characteristics of visible light quality [39]. Our study confirmed that RGB light resulted in the greatest accumulation of flavonoid contents in *R. hongnoensis* leaves, while the lowest accumulation was under B light. In contrast, Taulavuori et al. [39] found that G and B light increased the flavonoid content in tobacco leaves and red leaf lettuce. B LED exposure was also reported to increase the total flavonoid content of *C. paliurus* leaves by 37.7% and their quercetin content by 184.6% when compared to W LED after 60 days of treatment [40]. According to a study by Ouzounis et al. [41], the flavonoid content in tomatoes increased under 12% R supplementation, depending on the genotype. The high flavonoid content generated under RGB light conditions in the present study is believed to be due to the synergetic effect of R, G, and B light.

DPPH and ABTS were employed for the assessment of phenol content antioxidant capacity in the present work [42]. DPPH is a stable free radical that becomes a stable diamagnetic molecule by accepting electrons or hydrogen radicals [42]. In this study, the antioxidant activity of *R. hongnoensis* differed depending on light quality. Similarly, Shiga et al. [43] reported that the DPPH free radical scavenging activity of *Ocimum basilicum* L. was greater under R light than under B light. The antioxidant capacities determined via both methods (DPPH and ABTS) were in an agreement with the total phenol content of plants determined in our study. The present results are consistent with those of several other studies [44]. Chen et al. [45] reported a significant positive correlation between the total phenol content of persimmon and DPPH as well as ABTS radical scavenging activity. Therefore, it was concluded that the phenol contents of *R. hongnoensis* were increased under R light, thus improving antioxidant capacity.

## 4. Materials and Methods

### 4.1. Plant Materials and Growth Conditions

Germinating *R. hongnoensis* seeds (NIBRGR0000624110) provided by the National Institute of Biological Resources were used. Among the germinated seedlings, individuals with two or more true leaves were selected and transplanted into 72-cell plug trays with a commercial medium (Baroker; Seoul Bio Co., Ltd., Eumseong, Korea) and were grown under LED light for 8 weeks. After 4 weeks of LED light treatment, plants were transplanted into pots (10 cm in diameter), and after another 6 weeks, plants with a height of 8 cm or more were transplanted into pots (20 cm in diameter). During the experiment, air humidity in the cultivation room was maintained at 60%, the temperature was 23 °C, and the photoperiod was 12/12 h (dark/light), with tap water irrigation twice a week.

### 4.2. Light Treatment

**Figure 5:**
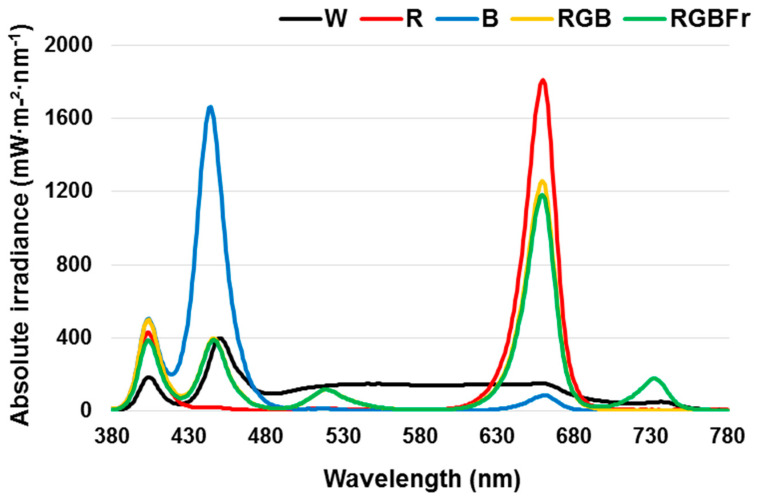
The spectral distribution of lights used in the closed walk-in growth chamber. Light quality used included W, white (as the control) light-emitting diodes (LEDs); R, red LEDs; B, blue LEDs; RGB, combined LEDs of R, green (G), and B at 7:1:2; and RGBFr, combined LEDs of R, G, B, and far-red (Fr) at 7:1:2:1.

The light source used in the experiment was an LED lamp (1280W, KNP LED, Daegu, Korea). Five types of single and mixed light sources were applied in different experimental treatment groups, including W light as a control, in addition to R (660 nm), B (444 nm), G (519 nm), and Fr (732 nm) LEDs. The light intensity was maintained at 200 µmol m^−2^ s^−1^ PPFD, and all treatments included 404 nm 20 µmol m^−2^ s^−1^ PPFD. The average PPFD was measured with a light meter (MK350, UPRTek, Jhunan, Taiwan) at a distance of 32 cm above the bench top. The spectral distribution of light in the experiment was measured using a spectroradiometer (MQ-200, Apogee, Logan, USA) 32 cm above the bench top, at 1-nm wavelength intervals. The spectral distribution and characteristics measured at three locations within the plant growing area for each treatment are shown in Figure 5. A completely randomized block design with 3 replications and 5 seedlings per treatment was used in the experiment. The treatment of locations in a controlled environment were randomly mixed between replications in order to minimize positional effects.

### 4.3. Growth Characteristics

Four weeks after treatment, plant height, leaf length, leaf width, leaf area, number of five-leaflets, root length, as well as the fresh and dry weights of shoots and roots were measured. Leaf area was measured using a leaf area meter (LI-3100, LICOR Inc., Lincoln, NE, USA), and the leaf area per compound leaf was determined. The number of five-leaflets was measured by counting the number of leaflets per compound leaf. Root length was measured as the length of the longest root. Fresh weight was measured using an electronic scale (CATY224, CAS Co., Seoul, Korea). Dry weight was measured after drying the tissues for 48 h at 70 °C in a drying oven (SJ-202DM, Sejong Scientific Co., Ltd., Bucheon, Korea).

### 4.4. Photosynthesis Measurements

Photosynthesis was measured in the terminal leaflet of the largest compound leaf using a portable photosynthesis system (Portable Photosynthesis system, Li-6800, LICOR Inc., Lincoln, NE, USA). The net photosynthesis, stomatal conductance, transpiration, and photosynthetic water use efficiency were calculated. Photosynthesis measurements were conducted 4 weeks after light treatment and immediately before final growth irradiation. Measurements were performed in survey mode and were repeated four times per treatment condition. The measurement conditions were 600 µmol·s^−1^ inflow air flow into the chamber, 25 °C temperature, 70% relative humidity, 3 cm^2^ leaf area, and 400 µmol mol^−1^ CO_2_. The light source in the chamber was removed to determine photosynthetic capacity under light conditions given to the experimental treatment.

### 4.5. Enzymatic Antioxidants

For the measurement of antioxidant activity, 100 mg fresh weight was added to 1 mL of 50 mM phosphate buffer with a pH of 7.0 containing 0.1 mM EDTA. The sample was extracted by bolting for 10 s and was then centrifuged at 4 °C and 13000 rpm for 20 min. One hundred milligrams fresh weight of *R. hongnoensis* were added to 1 mL of 50 mM phosphate buffer (pH 7.0) containing 0.1 mM EDTA, vortexed for 10 s, and centrifuged at 4 °C and 13,000 rpm for 20 min. SOD activity was measured via the method described by Alici and Arabaci [46]. SOD riboflavin was prepared by adding 2 g pvp, 50 µL triton-X, and 0.314 g riboflavin to 100 mL of 50 mM phosphate buffer. The SOD reaction mixture was prepared by adding 2 mM EDTA, 9.9 mM L-methionine, and 55 µM NBT to 100 mL of distilled water. In 1.25 mL of SOD reaction mixture, 50 µL of enzyme extract, and 200 µL of SOD riboflavin were mixed and reacted for 15 min under light at room temperature, and absorbance was measured at 560 nm. As a control, a reactant that was not irradiated with light was used.

CAT activity was measured with some modifications to the method described by Aebi [47]. The reaction mixture contained 100 µL of enzyme extract, 150 µL of H_2_O_2_, and 1.25 mL of 50 mM phosphate buffer. The change in absorbance at 240 nm was determined at 30-s intervals for 3 min. The molar extinction coefficient of H_2_O_2_ was [40 mmol^−1^ cml^−1^] at 240 nm.

GPX activity was measured as per the method described by Sadasivam and Manickam, with some modifications [48]. The reaction mixture contained 100 µL enzyme extract, 20 mM guaiacol 100 µL, 30 mM H_2_O_2_ 50 µL, and 1.25 mL 50 mM phosphate buffer. The change in absorbance at 436 nm was them measured at 30-s intervals for 3 min. The molar extinction coefficient of H_2_O_2_ was 25 mmol^−1^ cml^−1^] at 436 nm.

APX activity was measured as per the method described by Chen and Asada with some modifications [49]. The reaction mixture contained 100 µL of enzyme extract, 50 µL of 100 mM H_2_O_2_, and 1.3 mL of 50 mM phosphate buffer containing 0.6 mM ascorbic acid. The change in absorbance at 290 nm was measured for 33 min. The molar extinction coefficient of H_2_O_2_ was [2.8 mmol^−1^ cml^−1^] at 290 nm.

### 4.6. Extract Preparation

For the antioxidant assay, leaves, stems, and roots were harvested after 8 weeks of light treatment. The samples obtained by dividing the leaves, stems (shoots), and roots (roots) were frozen in liquid nitrogen and stored in a cryogenic freezer (UniFreezerU80, Daehan Scientific Co. Ltd., Wonju, Korea) at −80 °C. Frozen samples were ground in a mortar and used for the analysis. To prepare the sample extract, 100 mg of the sample and 1 mL of 50% methanol were mixed and stored for 6 h, followed by centrifugation (5424R, Eppendorf, Hamburg, Germany) at 13,000 rpm and 4 °C for 20 min.

### 4.7. Total Phenol Content and Flavonoid Content

The total phenol content of extracts was measured as per the Folin–Ciocalteu method [50]. To prepare the sample, 100 mg of the sample and 1 mL of 50% methanol were mixed and stored for 6 h, followed by centrifugation (5424R, Eppendorf, Hamburg, Germany) at 13,000 rpm and 4 °C for 20 min. Next, 500 µL of 2% Na_2_CO_3_ was added to a mixture of 450 µL distilled water, 250 µL 50% Folin–Ciocalteu solution, and 100 µL sample extract diluted 10 times, followed by incubation in the dark for 30 min. The absorbance was measured at 765 nm using a UV spectrophotometer (UV–1280, Shimadzu, Japan). The total phenol content was calculated using gallic acid as the standard.

### 4.8. Total Flavonoid Content

The total flavonoid content of the extract was measured according to the method of Kumaran and Karunakaran [51]. Fifty microliters of the extract was added to a mixture of 450 µL 80% methanol and 500 µL 2% AlCl_3_, vortexed for 2 s, and then reacted at room temperature for 30 min. After the reaction was completed, the absorbance at 415 nm was measured using a spectrophotometer. The total flavonoid content was calculated using quercetin as the standard.

### 4.9. DPPH Radical Scavenging Assay

The DPPH radical scavenging ability of the extract was determined according to the method of Blois [52]. DPPH was measured by adding 0.2 mM DPPH 800 µL to 200 µL of extracted sample, allowing it to react in the dark for 30 min, and then measuring the absorbance at 520 nm. Methanol was added instead of the sample extract as a control. Ascorbic acid was used as the positive control instead of the sample extract.

### 4.10. ABTS Radical Scavenging Assay

The ABTS radical scavenging ability of the extract was determined according to the method of Re et al. [53]. To prepare ABTS reagent, 7.4 mM ABTS and 2.6 mM potassium persulfate were mixed and stored in the dark for 24 h, and then the absorbance at 735 nm was adjusted to 0.7 ± 0.05. After adding 10 µL of the sample extract to 1 mL of ABTS reagent, the mixture was allowed to react for 10 min. Absorbance was measured at 735 nm. Methanol was added as a control instead of the sample. The scavenging activity (%) for cationic radicals and free radicals was calculated as [1−(sample/control)] × 100, and compared with L-ascorbic acid, a control material.

### 4.11. Experimental Design and Statistical Analysis

Data were analyzed for statistical significance using the SAS (Statistical Analysis System, V. 9.4, Cary, NC, USA) program. The experimental results were subjected to analysis of variance (ANOVA) and Duncan’s multiple range test.

## 5. Conclusions

R light treatment resulted in greater growth and promotion of antioxidant activity in *R. hongnoensis*. The results of this study demonstrated that R light increases the total phenol content as well as radical scavenging capacity. Further studies exploring the optimal light intensity and irradiation time are still needed in order to improve the application of R light technology for the promotion of *R. hongnoensis* antioxidant capacity.

## Figures and Tables

**Table 1:** The growth of *Rubus hongnoensis* under different light treatments on the 8th week after transplanting.

Light Quality ^z^	Plant Height (cm)	Leaf			No. of Five-Leaflets	Chlorophyll (SPAD)	Stem Diameter (mm)
		Length (cm)	Width (cm)	Area (cm^2^)			
W	6.8 b ^y^	13.4 a	8.4 a	44.7 ab	5.7 ab	38.0	5.3 a
R	11.2 a	16.7 a	10.4 a	66.2 a	6.6 a	40.7	6.6 a
B	3.7 c	8.8 b	5.5 b	24.1 c	5.1 b	34.3	2.8 b
RGBFr	7.9 b	15.0 a	8.5 a	51.6 b	6.8 a	39.6	5.2 a
RGB	8.2 b	15.3 a	9.0 a	55.2 ab	6.4 a	39.8	4.9 a
F-test	***	***	***	***	**	NS	**

^z^ Light quality included: W, white (as the control) light-emitting diodes (LEDs); R, red LEDs; B, blue LEDs; RGB, combined LEDs of R, green (G), and B at 7:1:2; and RGBFr, combined LEDs of R, G, B, and far-red (Fr) at 7:1:2:1. ^y^ Mean separation within columns by Duncan’s multiple range test at the 5% level. NS, **, ***: Non-significant or significant at *p* ≤ 0.01 or 0.001, respectively.

**Table 2:** Root length, fresh and dry weights of *Rubus hongnoensis* under different light treatments on the 8th week after transplanting.

Light Quality ^z^	Root Length (cm)	Fresh Weight (g)		Dry Weight (g)	
		Shoot	Root	Shoot	Root
W	27.1 bc ^y^	5.13 a	3.68 bc	0.95 ab	0.28 bc
R	33.1 ab	11.65 a	8.34 a	2.05 a	0.64 ab
B	25.2 c	2.76 b	2.06 c	0.35 b	0.12 c
RGBFr	34.4 ab	8.29 ab	7.98 ab	1.59 a	0.52 ab
RGB	38.4 a	11.90 a	8.59 a	1.94 a	0.66 a
F-test	**	**	**	**	**

^z^ See Table 1 for details on the light treatments. ^y^ Mean separation within columns by Duncan’s multiple range test at the 5% level. **: Significant at *p* ≤ 0.01.

## Data Availability

The authors will make the results available if requested.
